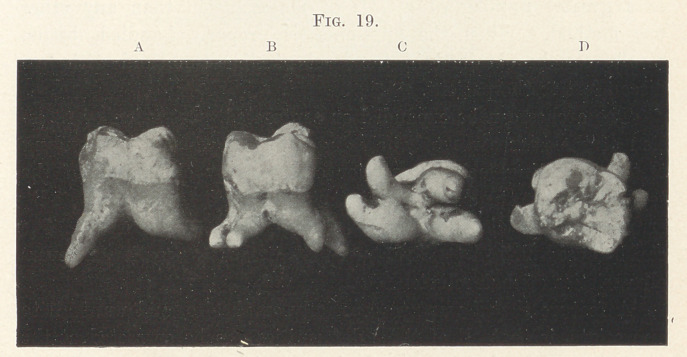# Impacted Teeth: Their Diagnosis, Liberation, and Extraction

**Published:** 1904-05

**Authors:** M. H. Cryer


### International Dental Journal.

Vol. XXV. May, 1904. No. 5.

### Original Communications.[1]

### IMPACTED TEETH: THEIR DIAGNOSIS, LIBERATION, AND EXTRACTION.[2]

BY M. H. CRYER, M.D., D.D.S.

This subject has been selected because of the wide diversity of opinion concerning the proper procedure in cases of impaction. The views which will be presented are based entirely on personal observations. While the conclusions to which these observations have led differ radically from those of many others in this field of work, the writer wishes to disclaim at the outset any intention or desire to criticise opposing views. He has no sympathy with the spirit which condemns without reservation methods based on experience, merely because they do not agree with one's own.

#### IMPACTED TEETH.

The term "impacted teeth" is generally used to designate a permanent tooth which has failed either wholly or partially to erupt. It is also sometimes employed to indicate the retarded eruption of a deciduous tooth.

---

[1] The editor and publishers are not responsible for the views of authors of papers published in this department, nor for any claim to novelty, or otherwise, that may be made by them. No papers will be received for this department that have appeared in any other journal published in the country.

[2] Read before the Academy of Stomatology, December 21, 1903.

Deciduous teeth, when impacted, are usually held in their developing capsule, which is covered by a dense fibrous gum tissue, the thickening being claimed by some observers to be due to the deposit of intercellular substance; by others to cell enlargement; by still others to the non-exfoliation of the older layers of the cells. Whatever may cause this tissue to become thickened and more dense is a problem which may be left to the pathologist.

In order to more clearly understand the anatomy and position of the teeth and their roots, a few illustrations will be introduced which the writer thinks may well be termed "Typical Anatomy."

Fig. 1 is a lateral view of a typical skull showing the teeth in relation with one another in an almost ideal position and occlusion.

Fig. 1.

Side view of a typical skull.

It is evident that there has been but little interference with the nutrition of either jaw or the teeth of this subject from the beginning of their development to the death of the subject. A little study of this illustration shows why, except in rare cases, the lower second molar should not be extracted in order to remove an impacted lower third molar. If the lower third molar be extracted only, the upper third molar is left without an antagonizing tooth; but if the lower second and third molars are both removed, the upper second and third molars have no antagonizing teeth.

Fig. 2 shows a jaw from which the external plates of the alveolar process have been removed, together with part of the

Fig. 2.

Antero-lateral view of upper and lower jaws, with the external plates of the alveolar process and some of the cancellated tissue removed.

cancellated tissue. The extraction of teeth from a jaw of this character would be comparatively easy, as the tissue is yielding and the tooth could be easily loosened and lifted out.

The writer so far has failed to find impacted teeth in jaws the cancellated tissue of which was in the typical condition shown in this illustration. One reason of this is that in jaws of this character, where there has been no interference with nutrition, the other teeth move forward to give proper room, as they are not held back by the cancellated tissue becoming dense and adherent to the cortical portion of the bone.

Fig. 3 is made from a section of the skull of a child about six years of age. The external plates of the alveolar process of the

FIG. 3.

Skull of a child about six years of age.

upper and lower jaws have been removed, exposing the deciduous teeth with their roots and developing permanent teeth, except the lower third molar and the upper first, second, and third molars. Parents of children often ask why teeth come in irregularly. Looking at this picture, in which the teeth are in normal position for a child of this age, one might rather ask, How do they ever get into their normal position at adult age? From experience alone it is known that these teeth will assume their normal positions, provided there is no interference with normal nutrition and no undue pressure from adjacent tissue. On the other hand, the least variation from normality in these respects will cause disarrangement of the teeth, the irregularity ranging from a slightly deflected incisor to an inverted molar, as shown in Fig. 12.

### ORDER OF IMPACTION.

The experience of the writer has been that the frequency of impacted teeth is as follows: First, the lower third molar; second, the upper canine; third, the upper third molar; fourth, the upper central incisor; fifth, the lower second premolar; sixth, the upper second premolar; seventh, the lower canine. The first and second groups of this classification will, without doubt, be accepted by all familiar with the subject under discussion. There are in the museum of the Dental Department of the University of Pennsylvania specimens as follows: Ten impacted lower third molars; nine impacted upper canines, two of which are in one jaw; two impacted upper third molars, both in the same jaw; two impacted central incisors; two impacted lower second premolars.

Examination of Fig. 3 makes apparent reasons for this order of impaction. It will be seen that the germ of the lower second molar is well back and partly within the ramus of the jaw. The germ of the lower third molar is still further upward and backward. As these teeth are developed and the jaw grows, the teeth and the cancellated tissue pass forward between the U-shaped cortical bone. If this sliding forward and downward of the tooth be interfered with by reason of inflammatory phenomena within the substance of the jaw, causing the cancellated and cortical portions to become adherent, the already erupted teeth will be prevented from yielding slightly to the eruptive force of the moving molar, and there will be no room for this tooth to slide into its proper position. The lower portion of the capsule is more liable to become retarded or fixed than the upper; consequently, in such a case the upper portion or crown of the tooth is carried forward and downward, causing it in many cases to take a horizontal position. In some instances it is turned directly upside down, as seen in Fig. 12.

If the position of the germ of the upper canine tooth be examined, it will be found at a higher level and deeper in the bone than the other teeth. The first premolar is erupted about three years before the canine, and often closes in towards the lateral, erupted five years previously, especially if the deciduous canine has been lost early. Under ordinary circumstances the canine will be forced into a fairly typical position, but if any inflammatory condition of the jaw has been manifested the bone may become firm and the canine more or less impacted. Similar conditions can be predicted of nearly all impacted teeth.

Occasionally supernumerary teeth may cause impaction. Fig. 4 shows a number of supernumerary teeth in the place which should

Fig. 4.

An odontoma and an impacted central incisor.

be occupied by a non-erupted left central. After the supernumerary teeth and a small portion of bone were removed, the true central tooth was located between the plates of bone forming the floor of the nose and the roof of the mouth.

Dr. Robert Huey, a member of this society, had a patient who had a similar impacted central incisor. Situated in front of it were some thirty-five small supernumerary teeth, which were removed, leaving the impacted tooth in its abnormal position, after which Dr. Huey succeeded in getting it into its proper place.

The case affords a good example of one of the methods by which the liberation of impacted teeth is accomplished. The writer has often found that where hard, dense bone prevented teeth from erupting into their proper position, upon the removal of this obstruction the teeth have passed into place, in some cases without mechanical aid, though usually this aid had to be extended.

THE REASON FOR THE LIBERATION OR EXTRACTION OF THE DECIDUOUS OR PERMANENT TEETH.

When a deciduous tooth is held beneath strong fibrous layers of gum tissue its growing roots extend in the direction of the blood- and nerve-supply, and their sharp edges cause irritation of the parts and through reflex action bring about various troubles. In such cases the crowns of these teeth must be set free. There is but one surgical operation justifiable,—*i.e.*, to cut the gum tissue in such a manner—varying somewhat in detail according to the shape of each tooth—as to liberate them from their prison. A deciduous tooth should be extracted when it is preventing a permanent tooth from taking its proper place. The non-performance of these duties at the proper time has more or less influence upon the position of the permanent teeth in adult life.

Permanent impacted teeth should, as a rule, be either liberated or extracted. When the impacted tooth can be brought into useful position through the extraction of supernumerary teeth, as in the case of Dr. Huey's patient, or by the removal of other causes impeding its eruption, the necessary steps for its liberation should be undertaken, whether the tooth be an incisor, canine, premolar, or molar. If left impacted, these teeth are liable to prevent the proper nourishment of other teeth, as shown in Figs. 9 and 15. They are also liable to interfere with healthy nutrition of the surrounding tissue as well. They may press upon the branches of the fifth pair of nerves, producing neuralgia, not only in the local region but in remote parts, and through reflex action they may cause various disturbances in and about the head and face. They are liable to bring about inflammatory conditions of this region, produce cellulitis in the tissues of the mouth, neck, throat, and the temporo-mandibular articulation, interfere with deglutition, etc. Then, again, parts of the roots may penetrate into the maxillary sinus or into the nasal chambers, as shown in Figs. 10 and 12, under which conditions, if they become devitalized, they are liable to infect these cavities.

INSTRUMENTS USED IN DIAGNOSIS AND EXTRACTING.

Fig. 5 gives an idea of some of the instruments used by the writer in diagnosing, liberating, and extracting impacted teeth. A shows the general shape of the excavator used as an exploring

FIG. 5.

G E C A

H F D B

Instruments used in diagnosing and extracting impacted teeth.

instrument. Small portions of bone may even be cut away with it until the crown is reached, and by a little manipulation the general direction of the crown and root could be usually diagnosed. B is a universal elevator. The blade is concavo-convex, and is long and sharp at the point. Its principal use is to loosen or dislodge a tooth by passing the thin blade between it and the adjoining tooth or between the tooth and the bone, with the concave portion next to the tooth. C and D are right and left elevators, which are especially useful in removing a root. E is a spiral osteotome used both as a drill and to cut bone laterally, or even to cut a portion of the tooth away. It is also used in removing bone which holds the tooth in a false position or prevents its removal. F is a surgical hand-piece. Both the osteotome and the handpiece are made very strong. The osteotome cuts with great rapidity when driven at full speed by the surgical engine. G is one of the most useful forceps the writer has used for extracting either upper or lower impacted teeth. H is a small forceps, similar to G, but used only for the lower teeth.

Many writers are very arbitrary in recommending instruments and methods of procedure. The instruments here shown are those which have been used by the writer for a long time, but he would not wish to criticise those who do not use them. Every man should use the tools he can handle best. The writer's method of diagnosing impacted teeth and their positions may also differ from that of others. Each man has his own way of doing these things, and he should do them in the way by which he can accomplish the best results.

### THE RADIOGRAPH AS AN AID IN DIAGNOSING THE POSITION OF IMPACTED TEETH.

The X-ray pictures have been of great service in locating foreign substances in various parts of the body, many of which could not have been located and removed without this assistance. Soon after the discovery of the X-ray for making skiagraphs of the human body, the writer gave considerable attention to the utility of skiagraphing the blood-vessels of the face for the study of their anatomy, also of impacted teeth as an assistance in diagnosis. In 1896 he wrote the following for the first edition of "The American System of Operative Dentistry:" "The diagnosis of unerupted teeth occupying abnormal positions has been greatly facilitated by special application of the newly discovered skiagraphic method." As a means of diagnosing the true position of impacted teeth, the method has not so far given the writer quite the same satisfaction as it does in general surgery. The position of an obscure impacted tooth renders it very difficult to get a good picture of the tooth with its anatomical relations to the neighboring structures; the cancellated tissue often becomes very dense from the same cause to which the impaction is due,—*i.e.*, malnutrition. Often only a slight shadow of the tooth shows in the picture. Even when a good shadow is obtained it is rather difficult to judge of the depth of the tooth in the bone. In other parts of the body pictures at right angles to each other can more readily be taken, so that if the foreign substance is indicated in both pictures the locality is much more easily established.

Great improvement has been made, however, in the past few years in obtaining radiographs of the jaws, and as this improvement advances the X-ray will doubtless become a more important aid in diagnosis.

After having seen some most beautiful stereoscopic radiographs, last July, in Europe, showing the internal anatomy of the brain-case, the writer thought that by making stereoscopic radiographs of the face, not only could the shape and size of various pneumatic sinuses and cells be diagnosed, but a much better idea of the position of impacted teeth could be given.

Through the kindness of Drs. Kassabian, Leonard, and Pancoast, of Philadelphia, the writer is enabled to show several skiagraphic pictures.

Fig. 6 is made from a radiograph taken by Dr. Pancoast. The permanent canine is missing from the arch. The patient did not lose the deciduous canine until after twenty years of age. Soon after the loss of the tooth a "bridge" was adjusted by being attached to the lateral and first premolar. This appliance is shown fairly well in the illustration; one can also see that the root-canal filling of the first premolar extends a little above the pin of the artificial tooth. The pulp-chamber and canal are also quite well shown in the second premolar. The permanent canine is distinctly visible in the picture, which also indicates that a portion of the crown is on the palatal side of the lateral incisor. Having carefully examined the patient's mouth before the picture was taken, the writer was able to make the same diagnosis as to the position of the tooth. It is possible that this tooth can be brought into position. Much, however, will depend upon the condition of the bone and the root. If the bone has become more than normally dense, and if the root has thickened or has curved,—the picture faintly indicates these conditions, especially the latter,—then it will be difficult to bring the tooth into position. At the time when this tooth should have made its appearance in the arch, a proper search made with an exploring instrument and the X-ray, and the removal of the deciduous tooth and whatever bone was holding it, would have permitted the tooth to advance, more than likely without other mechanical aid than guidance into its proper

Fig. 6.

Radiograph showing impacted canine tooth.

position. It is very interesting to note that the root of the first premolar is slightly curved backward. The second premolar is sensitive to percussion, which leads to suspicion that the impacted canine is interfering with the surrounding tissue.

ILLUSTRATIONS OF IMPACTED TEETH.

The various illustrations which follow have been selected to afford a good idea of the variable positions in which impacted teeth are found. They are taken from specimens in the museum of the Dental Department of the University of Pennsylvania.

Fig. 7 is made from a specimen owned by Professor James Truman. It shows a rather common form of impacted canine. In the living subject the diagnosis in this case would have been comparatively easy. In the first place, as the canine tooth would not have been found in the arch, the enlargement of the alveolar process over the impacted tooth would have indicated its position without much difficulty.

Occasionally there are cases of impaction of the canine and other teeth which do not produce external enlargements of the bone or gum tissue. The writer's experience has taught him that when the canine tooth is missing from the arch and it has not been extracted, the tooth lies somewhere within the jaw, though this is not always the case with the third molars and lateral incisors.

The writer has just had a patient about thirty-five years of age, from whose arch the two upper second premolars were missing, and who claims that he has not had them extracted. As he was suffering from neuralgia in the anterior portion of the maxillæ,

Fig. 7.

Fig. 8.

An impacted canine tooth, with the apex of the roots in the nasal fossa.

the writer thought that these teeth were impacted somewhere within the jaw, but careful exploration with instruments and radiographs taken at various angles and by different skiagraphers, failed to show any evidence of the missing teeth. The failure of these methods of examination leads the writer to believe that the teeth in question have never developed.

Fig. 8 is made from the nasal surface of the same specimen as is Fig. 7. It will be observed that the point of the root of the impacted canine is exposed on the outer wall of the nasal chamber. If this tooth should become diseased and an abscess form around the point of the root, the abscess would break into the nasal chamber.

Fig. 9 shows an impacted canine. It would be more difficult to diagnose the true position of this tooth than of those shown in

FIG. 9.

Impacted canine tooth.

FIG. 10.

Two impacted canine teeth.

Figs. 7 and 8. The crown would be easily located, as it was in the cadaver, even before the tissue dried, but the root, being embedded in the anterior wall of the antrum, would be difficult to locate.

Among numerous cases referred to the writer, was one sent to him by the late Professor Harrison Allen, who had been unsuccessfully treating the nose, and thought the teeth might possibly have something to do with the trouble. The crown of an impacted canine was easily located. The bone of the roof of the mouth had become hard and dense and adherent to the tooth. After removing the soft tissue and a portion of the bone, a fairly good hold could be taken of the crown, but the tooth could not be removed without danger of fracture. Fearing that damage might

Fig. 11.

An impacted upper third molar.

be done to the roof of the mouth, a small osteotome was attached to the surgical engine. The point was passed into the bone near the tooth, and as the osteotome would cut sidewise as well as penetrate, it was carried around the greater portion of the tooth until it could be loosened and removed. Afterwards treatment was comparatively easy, and the nasal trouble was easily cured.

Fig. 10 shows two impacted canine teeth. Their malposition caused the loss of the right second premolar, also the loss of the left first and second premolars. These teeth are in a rather common position for impacted canine teeth, though it is very unusual to have two in the same mouth. Their existence and position could easily have been diagnosed by an exploring instrument.

Fig. 11 shows an impacted upper third molar. A similar condition was found on the opposite side of the skull. In this case it would tax the powers of the radiographer to make a picture from the living subject which would reveal the true position of a tooth and roots when thus impacted.

The extraction of this tooth would be most difficult. When the mouth is thrown open the upper portion of the ramus of the jaw comes forward and interferes with the surgical work. The writer thus far has extracted all similar teeth that have been sent to him, leaving the second molar *in situ.* But they were not in so difficult a region as those shown in this picture. If teeth in similar positions do not interfere with the action of the mandible, or are not likely to produce a disturbance, such as abscesses or neu-

Fig. 12.

An impacted lower third molar.

ralgia, the writer would be inclined to let them remain in the jaw. But if they gave trouble, then they should be removed with as little injury to the surrounding tissue as possible. In rare cases it might be best to extract the second molar in order to reach the offending tooth, though every reasonable endeavor should be made to extract the third molar without disturbing the second.

Fig. 12 shows an impacted lower third molar turned completely upside down. Teeth of this character may give no trouble for years, or even be unsuspected, when, for some cause unknown, there may ensue a general inflammation of the surrounding tissue which might prevent proper movement of the mandible, interfere with deglutition, produce abscesses, neuralgia, etc. If such conditions should manifest themselves, and no other reasons could be found for this disturbance, then an impacted lower third molar should be suspected and a proper search made for it with the X-ray. The writer has diagnosed teeth in similar positions with proper exploring instruments, and, after finding them, has removed them with the aid of the surgical engine and forceps.

Fig. 13 shows a side view of two impacted lower third molars, the bone having been removed in order to expose the roots. It will be noticed that the anterior cusps are pressing against the concavity of the distal surface of the second molar. This condition makes these teeth most difficult to extract. The following plan

Fig. 13.

Side view of two impacted lower third molars.

has been adopted in a few cases: With a thin carborundum disk the anterior cusp has been cut away, then with an elevator the teeth have been turned out of their sockets. Occasionally these cusps are very deep down, as shown in Fig. 15, and are covered with gum tissue and situated below the level of the upper margin of the alveolar process, which makes it very difficult to cut off that part of the molar which is wedged in and against the second molar.

Fig. 14 is an interesting case of an impacted lower third molar, its position being on the inner side of the jaw, resting immediately upon the inferior dental nerves and vessels. It also rests partly below the line of the floor of the mouth and in close relation to the mylohyoid nerves and vessels. In extracting great care should be taken not to wound these.

Fig. 15 gives two views of an impacted lower third molar. A shows it in position, while B shows the tooth turned out of its socket. Part of the distal root of the second molar has been resorbed, exposing the root-canal, which more than likely caused pain and eventually the devitalization of the pulp. As the roots of the teeth are pressing in the region of the inferior dental nerve, it is possible that the function of the nerve was interfered with, which would probably cause neuralgia.

Fig. 14.

An impacted lower third molar.

An impacted tooth in this region would be somewhat difficult to diagnose, unless all the conditions are carefully studied, especially its position and relation to the second molar and surrounding tissues. In order to diagnose the tooth and its position all facts should be considered, such as the history, and the condition of the other teeth, especially those on the same side of the jaw. The patient, no doubt, would have had certain symptoms of disturbances. These facts should be ascertained, also the time of decay of the second molar, whether the third molar had been extracted, etc. A radiograph should be taken, as it would assist in confirming the diagnosis by other means that a tooth was impacted in this region. In examining the teeth in the lower jaw all were found to be in position except the lower third molar, which was not in view. When the patient was living he doubtless suf-

Fig. 15.

A

B

Two views of impacted lower third molar.

fered from neuralgia. The history of the case would have led one accustomed to close observation to suspect an impacted lower third molar. With the proper-shaped excavator, similar to that shown in Fig. 5, the sharp point could have been passed down through the gum tissue, immediately back of the second molar, to the enamel of the impacted tooth, a substance which cannot be mistaken by a trained dentist. There would be no difficulty in this, because the bony tissue would be porous, as it is in the specimen from which the illustration is taken. Sufficient bone could then be removed to give a general idea of the location of the tooth. The removal would be most difficult, as the entire tooth is so far down in the jaw, and the crown is well locked under and within the second molar. It would be possible with the surgical engine to remove the overlying bone until the tooth could be extracted. When a tooth is to be extracted it should be done with as little damage to the surrounding tissue as possible, and the extraction of another tooth, such as the second molar, in order to dislodge the third molar, should, if possible, be avoided.

In a living case like that shown in Fig. 15, if the patient were in distress, the writer would consider it good surgery to extract the second molar and allow the third molar to move forward, when it would more than likely be possible to remove the tooth without injury to that portion of the jaw. If the specimen be examined, it will be found that the bone on the lingual surface of the tooth is a mere shell, and that the bone below the tooth is so frail that it would fracture clear through if much pressure were put upon it. It is, in fact, so thin that the specimen has been broken through handling. Knowing the condition of this particular specimen, and having seen numerous fractures through the extraction of the lower third molar, the writer has been very cautious in such matters. He thus far has been fortunate in not having fractured a jaw, but he has seen cases of fractured jaws by thoroughly careful and competent surgeons.

As illustrating this caution regarding the extraction of the second molar under such circumstances, the writer remembers only one case where it seemed necessary to extract this tooth in order to relieve a disturbance caused by an impacted third molar. The late Professor Goodman, one of Philadelphia's well-known surgeons, called at the writer's office and asked him to come at once and bring his extracting instruments, as he had a patient on the verge of collapse. Upon examination a swelling was found near the angle of the jaw. The patient could open the mouth only a little way, and deglutition and respiration were difficult. In examining the parts with an excavator an impacted lower third molar was found. There was little time to be lost in relieving the patient, therefore the small lower forceps, G, shown in Fig. 5, were passed backward along the buccal cavity of the mouth, the inner beak passing between the upper and lower teeth until the lower second molar was reached, which was grasped in the beaks and extracted.

Two of the following illustrations are from X-ray pictures.

Fig. 16 is from a beautiful radiograph made by Dr. Kassabian. It shows two impacted lower third molars, which partly coincides

FIG. 16.

Radiograph showing two impacted lower third molars.

with the diagnosis made previously with the excavator. The history of the case is, that part of the crown of the left third molar has been broken away in an endeavor to extract the tooth, leaving the pulp exposed. The radiograph shows that the crown was deformed, also that the anterior cusp was apparently interlocked under the second molar. By careful examination with an excavator it was found that both of the anterior cusps were so far down in the tissues that the disk could not be used to remove them.

The patient being etherized, a mouth-gag was placed in position and a portion of the soft tissue removed with a small knife. The revolving spiral osteotome was placed within the broken crown or into the pulp-chamber, cutting almost through the balance of the crown. By passing the point of the osteotome under the crown and between it and the bone, a space was made partially in the tooth and partially in the bone, which allowed the point of the elevator B to pass between the tooth and the jaw.

The writer now seldom uses the forceps to remove a tooth after loosening it with the elevator. In using the elevator on the left side, as in this case, it is operated with the right hand, the surgeon standing on the left side of the patient. The left forefinger is placed in the mouth by the lingual side of the tooth and the thumb is placed on the buccal side of the first and second molars. This gives steadiness to the jaw and lessens the risk of slipping. As the tooth is raised from its socket the finger is placed so as to bring the tooth out of the mouth. If the tooth to be removed is on the right side, the elevator should be used with the left hand if possible (the surgeon standing on the right side). If the operator must use the elevator with his right hand, he should, however, manage to guard and steady the parts with his left hand.

Fig. 17.

Fig. 17 is made from three photographs of the tooth after extraction. A shows the outer or buccal side and its roots, in about the same position as when in the jaw. The distal cusps were broken away in a former endeavor to extract it. The greater portion of the crown was cut away with the surgical engine. On the side of the tooth there is a groove extending backward, downward, and inward, cut by the osteotome. It was along this groove that the elevator was forced under the tooth, causing the slight portion of the crown that remained to fracture. In B the tooth is turned slightly outward, in order to show three roots and the line of fracture which liberated the tooth. In C the tooth is turned upon its buccal surface, showing the two anterior cusps which were locked under the distal surface of the second molar.

Fig. 18 is from a good radiograph made by Dr. Leonard. The patient had some neuralgic trouble within the ear, and after having

Fig. 18.

Radiograph showing impacted upper third molar.

excluded several supposed causes, the teeth were suspected, as the upper first and second molars appeared to be sensitive, and this radiograph was taken. When one becomes accustomed to examining X-ray pictures, it is not difficult to detect a shadow of the crown of a tooth in the region where the upper third molar might be impacted, but one can get only a slight idea as to the depth of its occluding surface. No idea whatever is possible as to whether it is on an occluding line with the other teeth,—*i.e.*, whether it is near the buccal surface of the alveolar ridge or on the lingual surface. The roots of the tooth, their number, shapes, and positions, are not shown in the radiograph. All of this practical surgical diagnosis has to be learned by other means. In this case a careful exploration was made with an excavator, and the position of the crown was partially located. After the tissue covering the crown of the tooth had been cut away the tooth was grasped with the small forceps, G, shown in Fig. 5. The firmness of the tooth indicated that the roots were crooked and held by bone harder than normal. By carrying the handle of the forceps in the line of least resistance, which was outward, backward, and upward, the roots were unlocked from under the over-calcified bone.

Fig. 19 is made from four photographs of the tooth after extraction. A shows the anterior surface. B shows the distal sur-

FIG. 19.

A B C D

face, with the hook-like form of the buccal roots. C shows the upper surface, or the root end of the tooth, with the four roots spread outward, approaching a horizontal direction, and D shows the occluding or grinding surface, with the points of the roots extending outward. It will be noticed that this tooth is quite a different object from that shown in the skiagraph. It may be interesting to know that the ear has improved since the extraction, and at the same time the other molars appear to have lost their sensitiveness, indicating that the tooth was interfering with the nerve supplying these teeth.

## Figures and Tables

**Fig. 1. f1:**
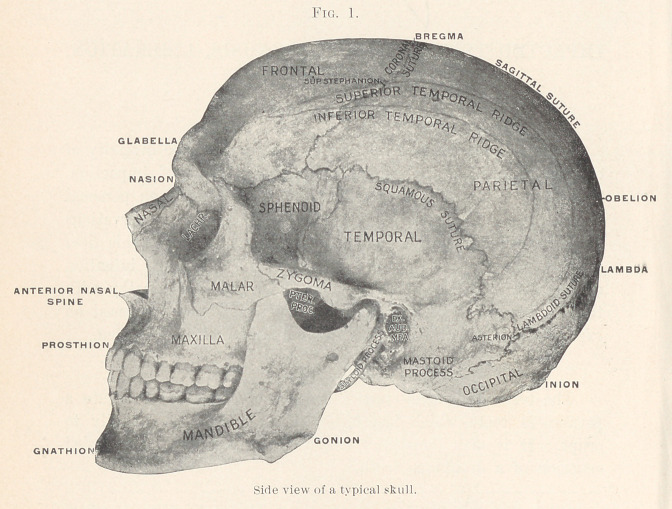


**Fig. 2. f2:**
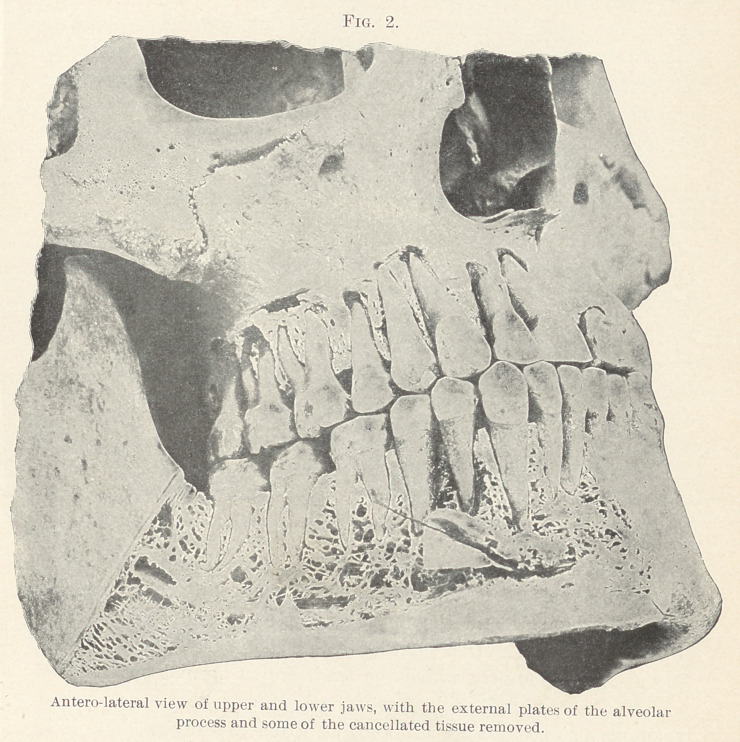


**Fig. 3. f3:**
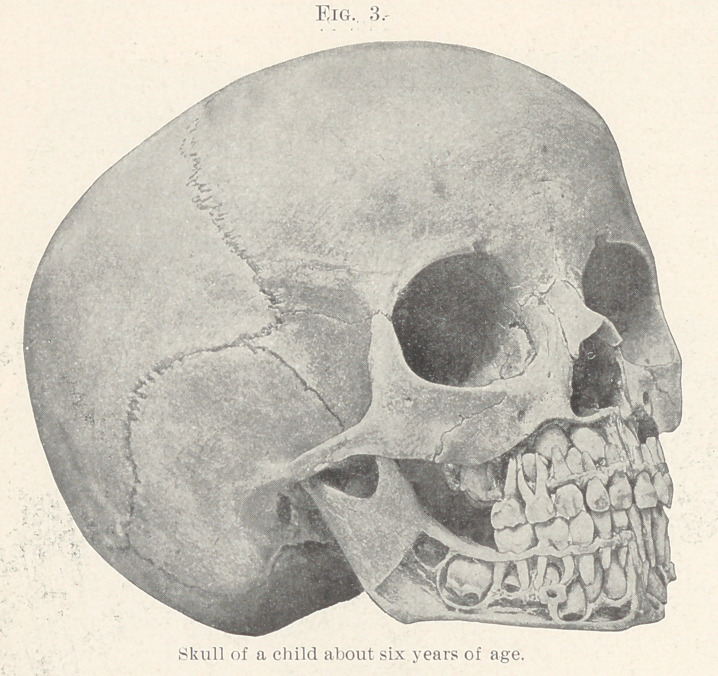


**Fig. 4. f4:**
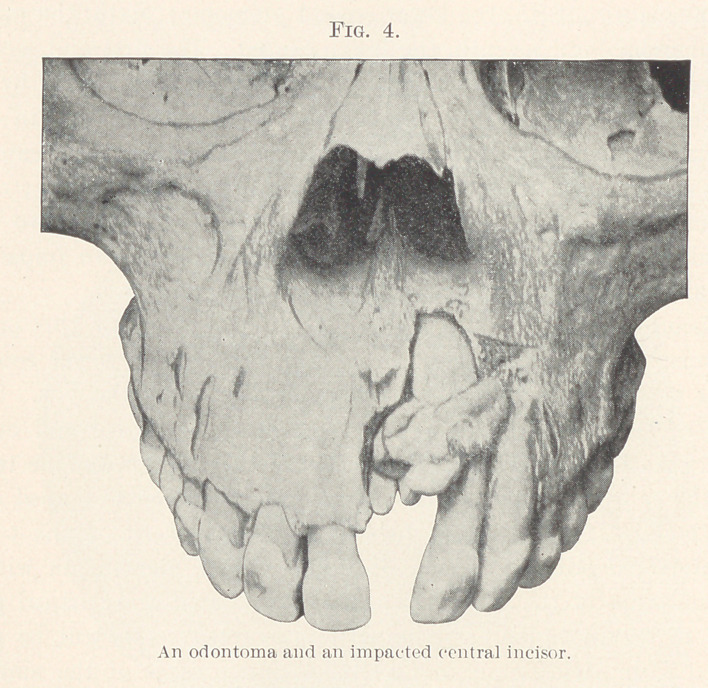


**Fig. 5. f5:**
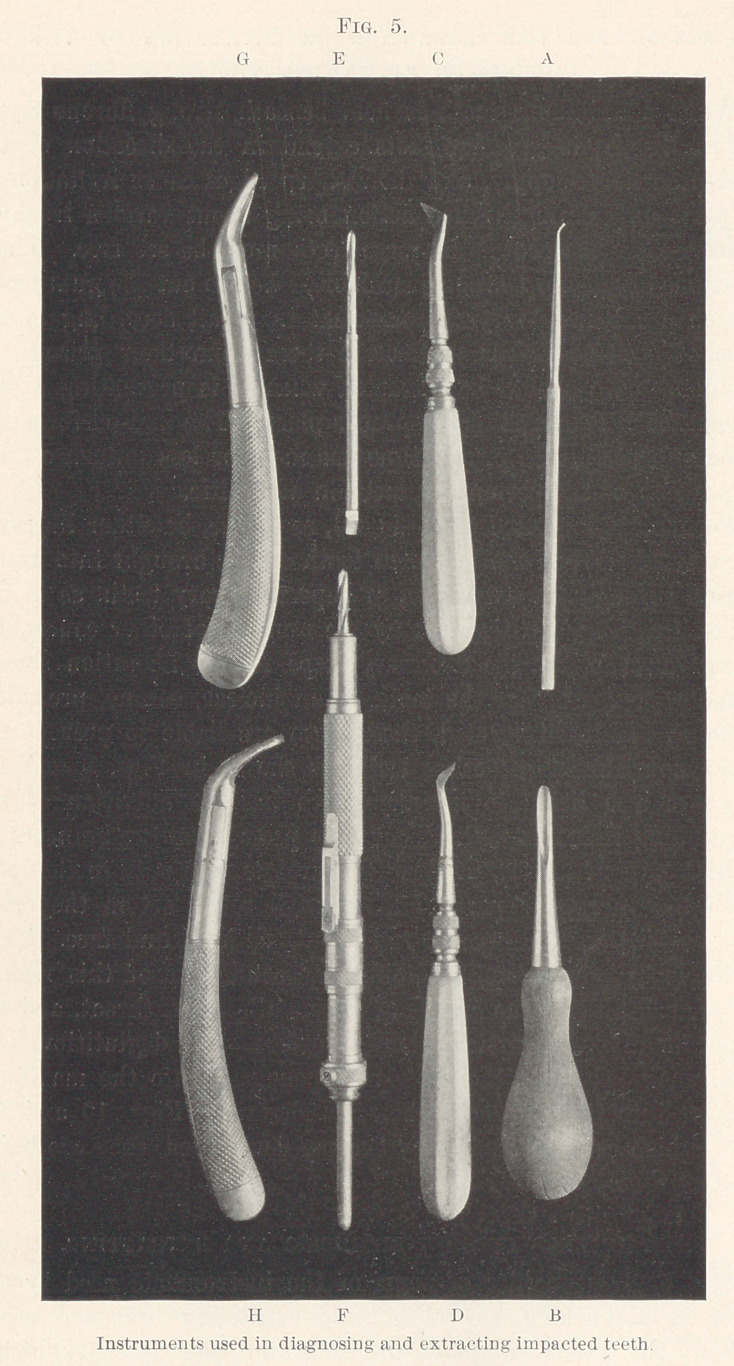


**Fig. 6. f6:**
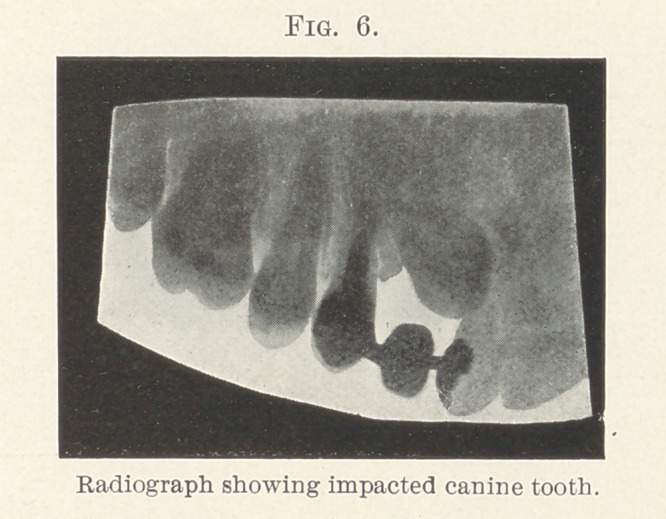


**Fig. 7. Fig. 8. f7:**
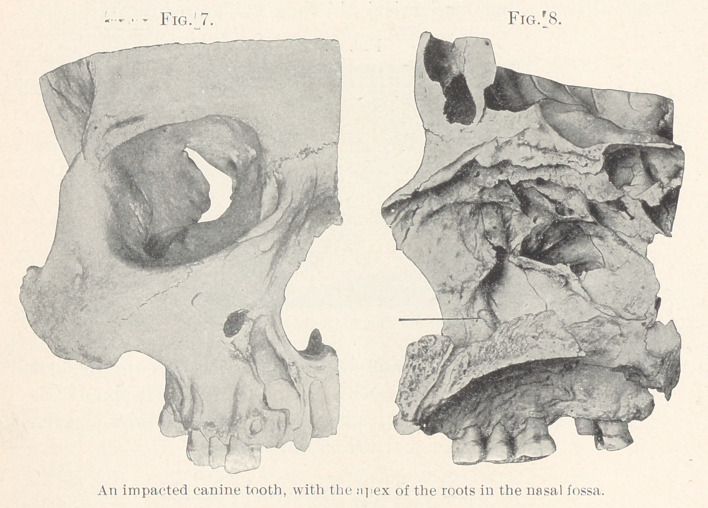


**Fig. 9. f8:**
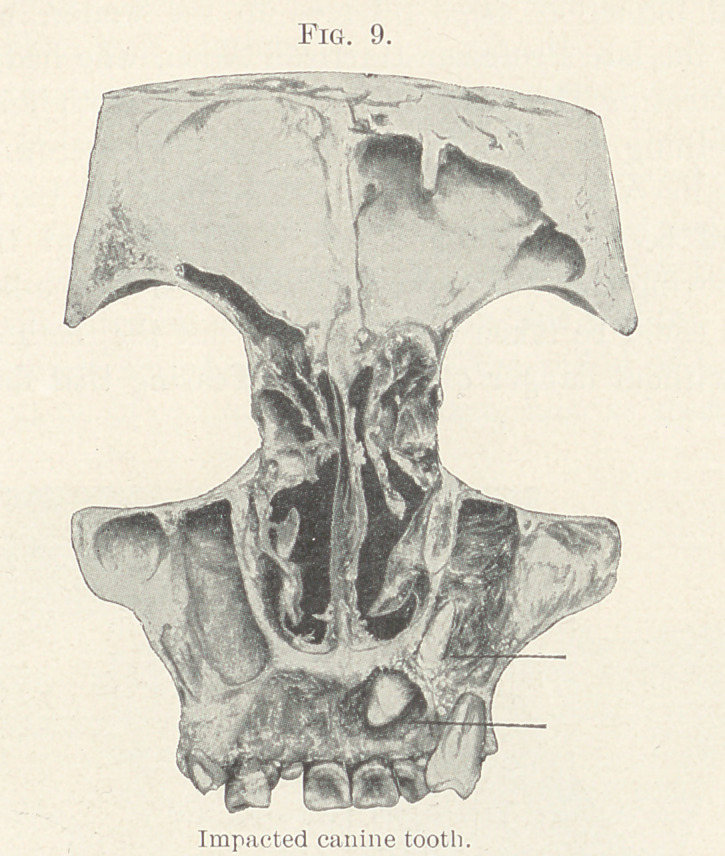


**Fig. 10. f9:**
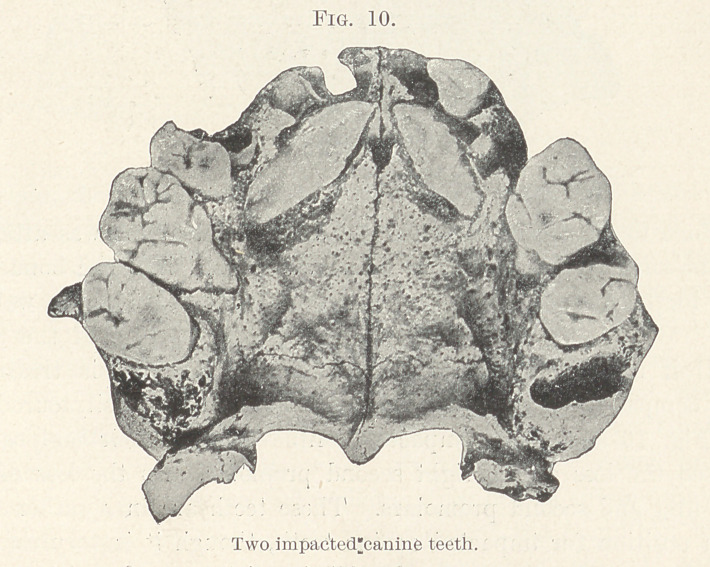


**Fig. 11. f10:**
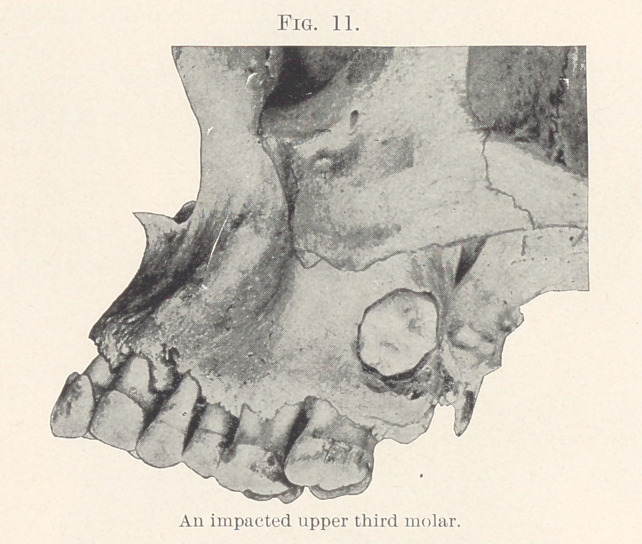


**Fig. 12. f11:**
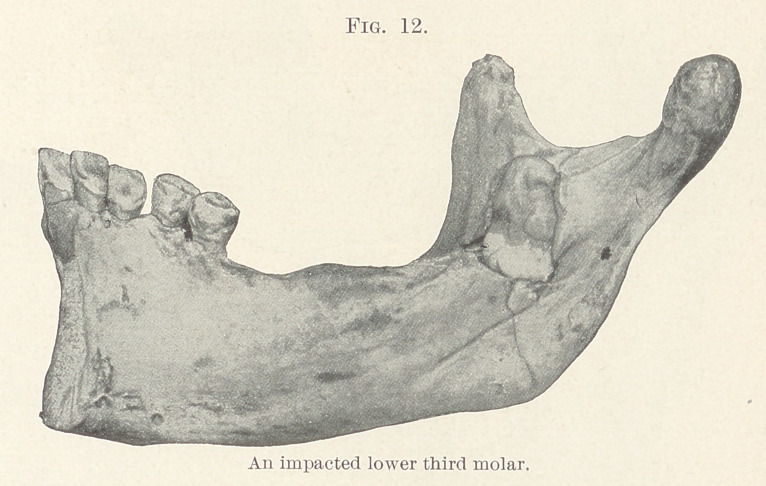


**Fig. 13. f12:**
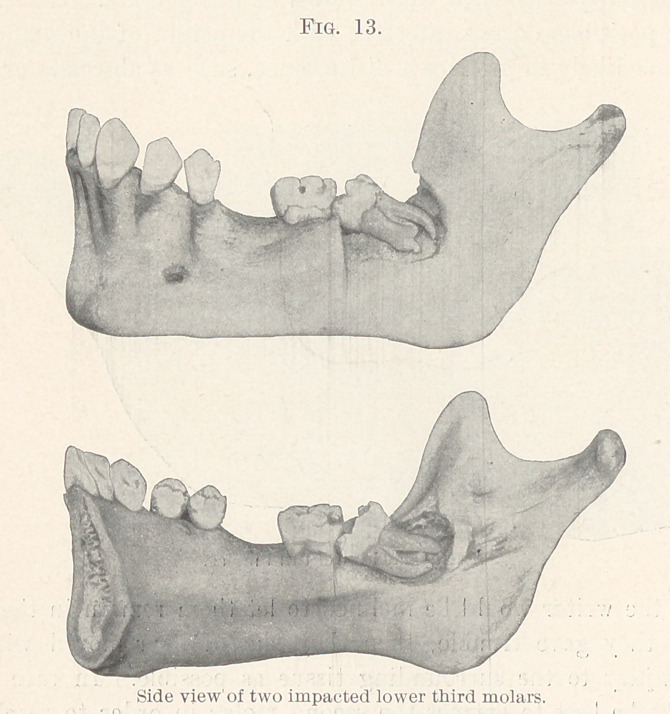


**Fig. 14. f13:**
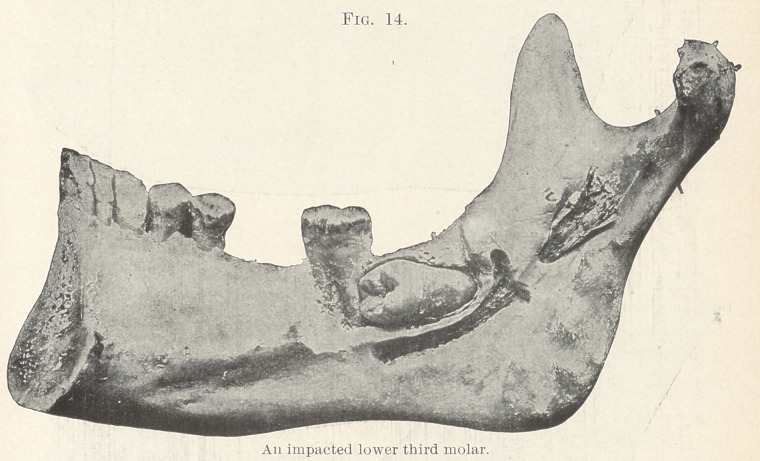


**Fig. 15. f14:**
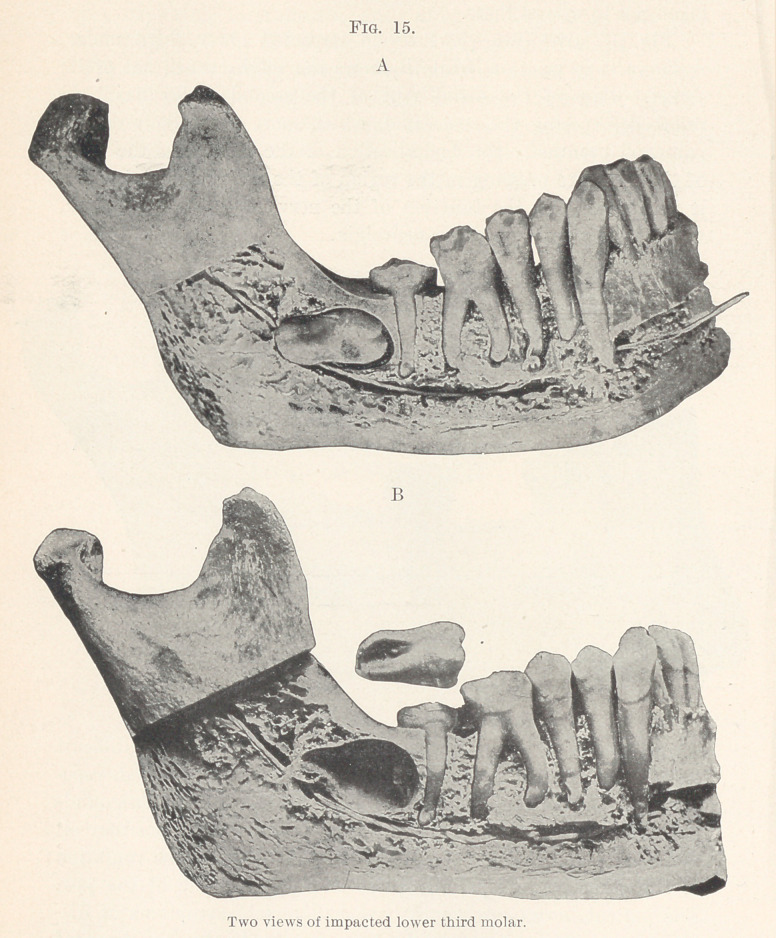


**Fig. 16. f15:**
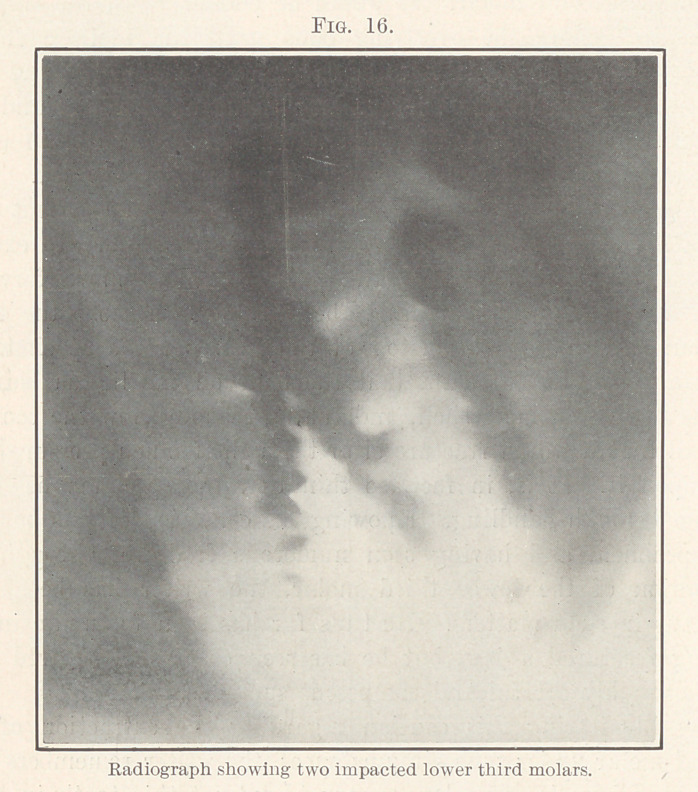


**Fig. 17. f16:**
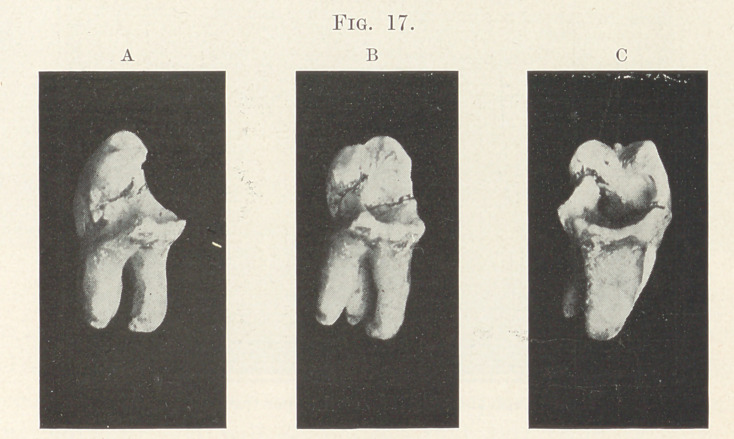


**Fig. 18. f17:**
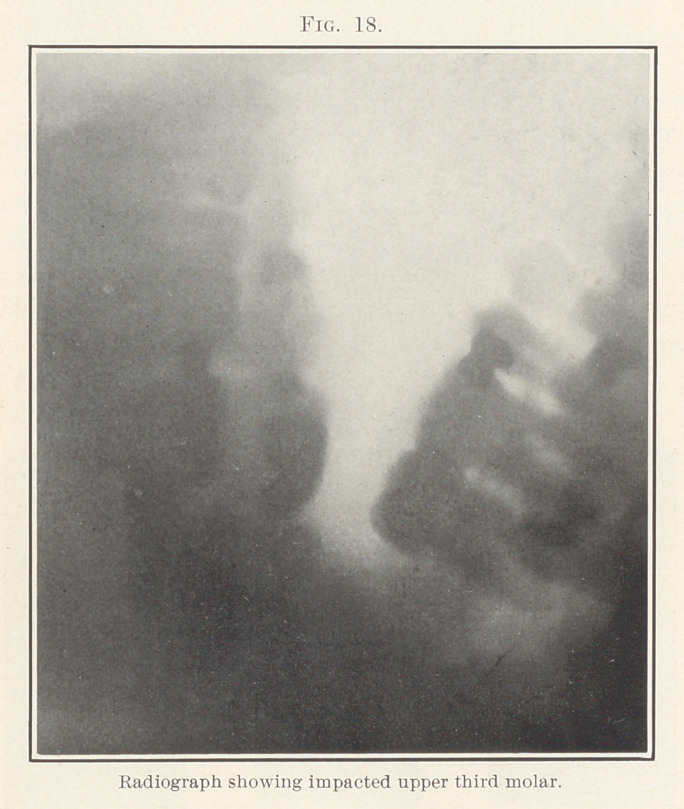


**Fig. 19. f18:**